# IL-6 Levels Influence 3-Month All-Cause Mortality in Frail Hospitalized Older Patients

**DOI:** 10.14336/AD.2020.0713

**Published:** 2021-04-01

**Authors:** Stefano Rizza, Pasquale Morabito, Livia De Meo, Alessio Farcomeni, Giulia Testorio, Marina Cardellini, Marta Ballanti, Francesca Davato, Chiara Pecchioli, Giovanni Di Cola, Maria Mavilio, Massimo Federici

**Affiliations:** ^1^Department of Systems Medicine, University of Rome Tor Vergata, Rome, Italy; ^2^Department of Economics and Finance, University of Rome Tor Vergata, Rome, Italy

**Keywords:** in-hospital mortality, IL6, MPI, albumin, frailty, elderly

## Abstract

The multidimensional prognostic index (MPI) is a sensitive and specific prognosis estimation tool that accurately predicts all-cause mortality in frail older patients. It has been validated to assess the risk of 1-month to 2-year mortality in frail older patients during hospitalization and after hospital discharge. However, whether the MPI is a valid prognostic tool for follow-up periods of different lengths remains to be validated. To this end, we followed up 80 hospitalized patients (female=37, male 43) at least 75 years of age (mean age=82.6±4.4, range=75-94 years) to assess the 3-month all-cause mortality (mean follow-up=61.0 ± 31.7 months [range 4-90 days]). Accordingly, patients were subdivided into low (MPI-1, score 0-0.33), moderate (MPI-2, score 0.34-0.66) and high (MPI-3, score 0.67-1) mortality risk classes. Moreover, baseline biochemical, inflammatory and metabolic parameters, as well as anamnestic and clinical characteristics, were obtained. Although the MPI-3 score was significantly associated with 3-month all-cause mortality in univariate analysis (HR=5.79, 95%CI=1.77-18.92, p=0.004), a multivariate model indicated that only low albumin (HR=0.33, 95%CI=0.16-0.68, p=0.003) and high IL6 (HR=1.01, 95%CI=1.00-1.02, p=0.010) levels were significantly associated with 3-month all-cause mortality. In conclusion, we suggest that measurement of IL6 as well as albumin, rather than the MPI score, may help in providing tailored therapeutic interventions to decrease short term mortality in older hospitalized individuals.

As the aging population increases rapidly worldwide, caring for frail older adults has become mandatory in modern medicine (1). Indeed, older adults account for almost 50% of all inpatient hospitalization days (2); therefore, correct identification of prognostic tools to facilitate tailored therapeutic interventions in aged individuals is a public health priority (3). In fact, hospitalization for an acute medical event can be traumatic and harmful in older people and can often lead to several important clinical complications, such as functional decline and death, which are frequently unrelated to the complaint at admission (4). Therefore, the multidimensional prognostic index (MPI), a validated and accurate algorithm for assessing the risk of 1-month to 2-year mortality, is used to assess frail older patients after hospital discharge (5-8). The MPI is based on Comprehensive Geriatric Assessment (CGA) comprising six common geriatric scales for cognitive, functional, nutritional and clinical status, as well as on information about drugs taken and patients’ social support (9). However, from a biochemical viewpoint, a typical signature of aging is widespread changes in protein expression and secretion, a condition referred to as inflammaging, which is characterized by a broad range of chemokines and bioactive molecules including C reactive protein (CRP), IL-1β, TNF-α and IL-8. Among them, increased expression of IL-6 has been reported to be a robust predictor of disability and frailty (10) as well as cardiovascular and all-cause mortality in the general older population (11). Consequently, interest has increased in the clinical use of interactions between biomarkers and multidimensional geriatric assessment to identify older patients at higher risk of frailty and death (12-14). Here, we performed a hypothesis-generating short follow-up to investigate 3-month total mortality after discharge in a group of very old inpatients hospitalized for acute and severe medical events.

## MATERIALS AND METHODS

### Participants

From February to July 2019, we conducted a prospective observational study following the Declaration of Helsinki and the guidelines for Good Clinical Practice. The study was approved by the local hospital Ethics Committee. We included hospitalized patients admitted for acute disease or relapse of a chronic disease; all patients were admitted to the emergency department and were then moved to the internal medicine department. Participants were eligible if they were at least 75 years of age and were able to provide informed consent and complete a standardized CGA to calculate the MPI at hospital admission. They were followed for 3-month total mortality beginning from the day of hospital discharge. Exclusion criteria included the presence of end-stage neoplastic disease, end-stage chronic kidney disease (e-GFR ≤15 ml min^-1^ [1.73 m]^-2^, according to the Chronic Kidney Disease Epidemiology Collaboration [CKD-EPI] formula), any connective tissue or bowel inflammatory diseases and sepsis. We also recorded the main diagnosis at discharge, coded according to the Italian translation of the International Classification of Diseases, 9th revision, Clinical Modification (ICD-9-CM).

Body mass index (BMI) was calculated by dividing the weight (in kilograms) by the square of the height (in meters). Blood pressure was measured in the dominant arm with a standard appropriately sized sphygmom-anometer cuff. Current and former smokers were considered as a single group and compared with people who were never smokers. Approximately 30 ml whole blood samples were obtained between 6:00 and 7:00 AM on the first admission day after an overnight fast.

### Multidimensional prognostic index (MPI)

The MPI is an algorithm developed and previously validated in older hospitalized patients that efficiently predicts 1-year mortality (6). Briefly, the MPI was developed to include information from the eight domains of the CGA: Activities of Daily Living (ADL), Instrumental Activities of Daily Living (IADL), the Short Portable Mental State Questionnaire (SPMSQ), Cumulative Illness Rating Scale (CIRS), Mini-Nutritional Assessment (MNA), Exton-Smith Scale (ESS), medication use and cohabitation. The sum of the calculated scores from the eight domains is divided by eight to obtain a final MPI risk score between 0=no risk and 1=higher risk of mortality. As previously reported (6), the MPI is expressed as three grades of risk: MPI-1, low risk (MPI value ≤0.33); MPI-2, moderate risk (MPI value between 0.34 and 0.66); and MPI-3, severe risk (MPI value >0.66).

### Cytokine assays

In the serum, cytokine expression levels were determined with Simple Plex, an integrated immunoassay system for rapid and sensitive detection of as many as four targeted protein antigens across multiple biological sources. Simple Plex assays consisting of a disposable microfluidic cartridge and an automated analyzer, the Ella instrument, were performed according to the manufacturer’s instructions (Protein Simple, CA, USA). Briefly, human serum samples were diluted 1:2 for IL-1β, IL-6 and TNF-α measurements in sample diluent SD13; diluted samples, quality control samples and buffer were loaded into each cartridge. A barcode scanner was used to identify the cartridge and automatically load lot-specific factory-calibrated standard curves embedded in each cartridge barcode. Sample identities and dilution factors were input with Simple Plex Runner software. At the conclusion of the assay, triplicate results (one per glass nano reactor [GNR]) for every analyte in each sample were automatically displayed. Raw (background-subtracted) signal levels were reported in relative fluorescence units (RFU) for each GNR, and the mean RFU signal values, standard deviation and coefficient of variation are provided for triplicate GNRs. RFU values were automatically back-fitted to barcode-embedded standard curves, and back-fitted concentrations were multiplied by user-defined dilution factors to provide calculated concentrations in pg/ml for each analyte for every sample.

### Statistical analysis

Quantitative data are expressed as mean±SD, whereas categorical variables are expressed as number (percentage) of participants. Correlations between continuous variables were estimated with Spearman correlation. Time-to-event data were analyzed with log-rank test or univariate Cox-models for categorical or continuous variables, respectively.

To identify a multivariate Cox regression model, we set the maximum number of predictors to two, according to a common heuristic rule of having approximately one predictor per 15 events. A best subset approach was used, wherein all possible multivariable Cox regression models with two predictors were compared. The best one, selected with the Akaike information criterion, ultimately included albumin and IL6. Of note, the same model was chosen with a forward stepwise selection on the basis of AIC. All variables considered in univariate analysis were candidates for the multivariable Cox regression. All p-values are two tailed, and the significance level was fixed at 5% before the analysis. All analyses were conducted in R (R Development Core Team, Vienna, Austria) software version 3.5.1.

**Table 1:** Baseline clinical and laboratory characteristics of patients divided according to follow-up mortality. Data are shown as numbers or mean (±SD).

Parameter	Overall (n=80)	All-cause mortality (n=24)	Survivors (n=56)	p
Age (years)	82.3±4.4	83.4±4.0	83.0±4.3	0.285
Sex (m/f)	43/37	11/13	32/24	0.246
BMI	25.2±4.4	23.4±3.7	25.3±4.4	0.086
Albumin (mg/dl)	3.2±0.6	2.8±0.5	3.3±0.5	<0.001
Fasting glucose (mg/dl)	124.1±56.2	138.8±69.7	117.8±48.7	0.128
Fasting insulin (mU/L)	10.6±12.7	10.4±18.7	10.7±9.7	0.931
HOMA-IR	3.1±3.9	3.1±5.3	3.1±3.2	0.981
HbA1c (mmol/mol)	45.8±14.9	46.4±15.0	45.5±15.0	0.801
Total cholesterol (mg/dl)	151.1±46.5	132.2±56.3	159.2±39.5	0.040
HDL cholesterol (mg/dl)	34.8±15.3	29.3±15.4	37.2±14.8	0.039
Triglycerides (mg/dl)	112.8±40.7	102.1±36.7	117.3±41.7	0.109
Hgb (g/dl)	11.4±1.9	11.0±2.0	11.6±1.8	0.233
White blood cells (n/mm³)	10.3±4.9	11.1±5.5	9.9±4.6	0.344
Hypertension (yes/no)	62/18	17/7	45/11	0.256
Diabetes (yes/no)	27/53	9/15	18/38	0.414
CV diseases (yes/no)	49/31	14/10	35/21	0.457
Neurodegenerative diseases (yes/no)	18/62	5/19	13/43	0.566
Pressure ulcers (yes/no)	9/71	5/19	4/52	0.086
Previous cancer (yes/no)	13/67	6/18	7/49	0.145
Urinary catheter (yes/no)	27/53	11/13	16/40	0.109
E-GFR (<50 ml/min/≥50 ml/min)	44/36	16/8	28/28	0.129
Follow-up (days)	61.0±31.7	32.9±4.1	83.0±15.4	<0.001
Hs-CRP (mg/dl)	55.4±57.7	66.9±63.9	50.4±54.7	0.244
TNF-α (ng/ml)	20.5±17.5	23.2±22.0	19.4±15.2	0.436
IL-1β (ng/ml)	0.32±0.70	0.47±1.01	0.25±0.49	0.210
IL6 (ng/ml)	24.6±32.5	41.8±45.4	17.3±21.7	0.002
MPI classes (1/2/3)	34/28/18	4/11/9	30/17/9	0.007
Statin therapy (yes/no)	23/57	7/17	16/40	0.144
Main diagnosis at discharge	
Anemia (yes/no)	8/80	2/24	6/56	n.s.
Late-stage cirrhosis	6/80	2/24	4/56	n.s.
Coronary artery diseases	11/80	3/24	8/56	n.s.
Heart failure	8/80	2/24	6/56	n.s.
Pneumonia	24/80	8/24	16/56	n.s.
Syncope	8/80	2/24	6/56	n.s.
Renal failure	15/80	5/24	10/56	n.s.

## RESULTS

**Figure 1.**
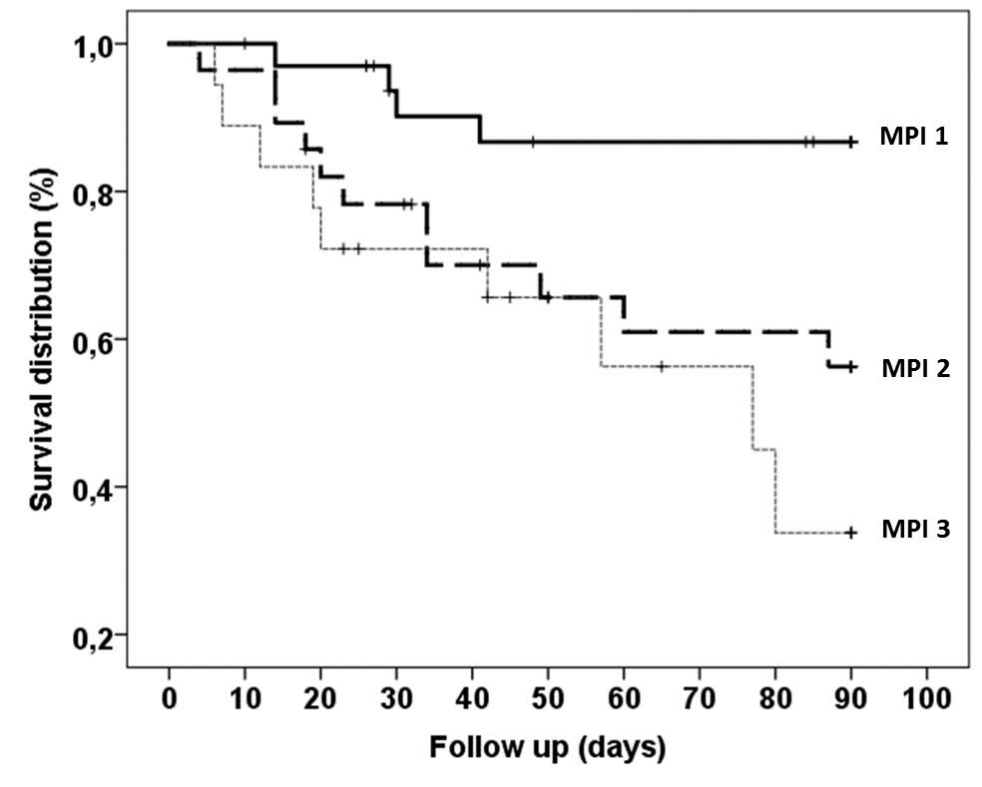
Survival curves of 3-month all-cause mortality for different grades of the multidimensional prognostic index (MPI) (Χ²=10.8, p=0.004). MPI 1=low risk; MPI 2=moderate risk; MPI 3=severe risk.

During the enrollment period, 97 patients were screened for inclusion. However, six patients were excluded because they were younger than 75 years, and 11 patients were excluded because the CGA was not completed. Therefore, the final follow-up included 80 patients (43 men and 37 women, with a mean age=82.6±4.4 years and range=75-94 years). During follow-up (61.0±31.7 days), 24 deaths occurred, corresponding to an overall 3-month mortality incidence rate of 30%. As expected, (Fig. 1), individuals in the MPI-3 group died more frequently than those in the other two groups (Χ²=10.8, p=0.004). Accordingly, those who died during follow-up more frequently had higher MPI risk classes (Table 1, p=0.007). Furthermore, they tended to have lower BMI levels and to have pressure ulcers (Table 1, p=0.086 for both) and significantly lower albumin and total and HDL cholesterol levels (Table 1, p<0.001, p=0.040 and p=0.039, respectively) than those surviving at follow-up. In contrast, the two groups had similar rates of diabetes, hypertension, history of cardiovascular diseases, previous cancer, urinary catheter use, and glucose and insulin levels. Interestingly, among inflammatory markers, only IL-6 was statistically significantly higher in individuals who had died by the follow-up than in other individuals (p=0.002). No other inflammatory markers differed between groups. IL-6 significantly correlated with CRP (r=0.273, p=0.014) but not with IL-1β, TNF-α and WBC. The best multivariable Cox regression model indicated that the 3-month all cause-mortality was significantly and independently associated with high IL6 levels (HR=1.01, 95%CI=1.00-1.02, p=0.010) and low albumin levels (HR=0.33, 95%CI=0.16-0.68, p=0.003). The MPI score was excluded from the Cox model, although it was significantly associated with study outcome in univariate analysis.

**Table 2:** Univariate and multivariate analysis models for 3-month all-cause mortality.

	Univariate hazard ratio (CI 95%)	p	Multivariate hazard ratio (CI 95%)	p*
Age (y)	1.05 (0.96-1.14)	0.2752		
Sex (male)	1.53 (0.68-3.42)	0.30		
BMI	0.92 (0.84-1.02)	0.108		
Albumin	0.26 (0.13-0.52)	<0.001	0.33 (0.16-0.68)	0.003
Fasting glucose	1.01 (1.00-1.01)	0.044		
Fasting insulin	0.99 (0.96-1.04)	0.91		
HOMA-IR	1.00 (0.89-1.13)	0.9933		
HbA1c	1.01 (0.98-1.03)	0.641		
Total cholesterol	0.99 (0.98-0.99)	0.005		
HDL cholesterol	0.96 (0.93-0.99)	0.014		
Triglycerides	0.99 (0.98-1.00)	0.143		
Hgb	0.87 (0.70-1.09)	0.227		
White blood cells	1.04 (0.96-1.13)	0.281		
Hypertension	0.68 (0.28-1.63)	0.385		
Diabetes	1.35 (0.59-3.08)	0.488		
Cardiovascular diseases	0.86 (0.38-1.94)	0.729		
Neurodegenerative diseases	1.10 (0.45-3.88)	0.662		
Pressure ulcers	2.80 (1.04-7.52)	0.0413		
Previous cancer	1.71 (0.68-4.30)	0.264		
Urinary catheter	1.82 (0.81-4.06)	0.145		
E-GFR (<50 ml/min/≥50 ml/min)	1.94 (0.83-4.54)	0.135		
CRP	1.01 (0.99-1.01)	0.126		
TNF-α	1.01 (0.99-1.03)	0.313		
IL-1β	1.18 (0.81-1.72)	0.388		
IL6	1.02 (1.01-1.03)	<0.001	1.01 (1.00-1.02)	0.010
MPI classes (2 vs 1)	4.05 (1.29-12.73)	0.017		
MPI classes (3 vs 1)	5.79 (1.77-18.92)	0.004		

## DISCUSSION

Substantial literature and calls for action have focused on ameliorating the management of older adults in terms of frailty control, quality of life improvement and better care regarding multi-morbidity (15-16). Accordingly, in this prospective study, we found a significant association between elevated levels of IL6 and greater 3-month all-cause mortality in older patients hospitalized for acute disease or relapse of chronic disease. Overall, our study confirms the relevance of IL-6 as a valuable and clinically meaningful biomarker in geriatric medicine (17). In fact, the literature has reported that IL-6 concentrations significantly predict both subclinical (sarcopenia and insulin resistance) (18-19) and clinical (disability and mortality) (20) conditions in older people. Therefore, our main finding reinforces the clinical role of IL-6 as a prognostic indicator, thus suggesting that this proinflammatory cytokine is associated with mortality independently of the presence of other common clinical conditions and disease risk factors. In fact, the strength of the association that we observed between IL-6 and mortality was not significantly modified by the inclusion of potential confounders in the adjusted models. Actually, several studies reported an increased level of IL-6 also in other different clinical settings such as sedentary behavior, obesity, diabetes, cardiovascular diseases, cancer, and recently in COVID-19 patients with poor outcomes, all characterized by a high mortality rate.

Notably, our findings are consistent with the controversial results regarding the role of IL-1β in aging. Although IL-1β may be considered a reliable inflammatory biomarker of physiological decline, some reports have found that IL-1β is downregulated in frailty and sarcopenia. Moreover, because IL-1β is upstream of the pathway that induces IL-6 secretion in the immune response (31), our results may indicate that an overload of IL6 release may result from tissue injuries or inflammation sites partly free of IL-1β control. We believe that this point is of particular interest, because short-term mortality in hospitalized older patients is often high, and an appropriate and simple biochemical test might support patient management during hospital stays and early after discharge.

Although in univariate analysis the MPI score was significantly associated with 3-month all-cause mortality, our statistical model excluded this variable from the multivariate examination, in contrast to many scientific studies reporting that it is a robust prognostic factor in various geriatric clinical settings (21-22). However, the limited number of study participants might have affected the predictive efficacy of the MPI score. Another possible explanation may be the very short length of this study’s follow-up, which may have made the MPI inappropriate for the study outcome. Our findings must also be considered in light of the MPI having been designed as a predictive tool for 1-year mortality. Future perspective studies including a larger number of participants are therefore warranted to understand whether the MPI score remains a valid prognostic tool for follow-up periods of different lengths. Similarly, the presence of diabetes, hypertension, previous cardiovascular diseases, cancer, decreased renal function, pressure ulcers and urinary catheters did not affect the risk of mortality.

Notably, albumin is often a good marker of nutritional status rather than a robust mortality risk factor, because there is no clear evidence that a reduction in albumin levels to pathological values inevitably occurs with age. In fact, in people in clinical stable condition, albumin often remains in a normal range even after the age of 90 (23). Our results clearly indicated that low albumin levels are a significant predictor of 3-month all-cause mortality in older hospitalized individuals. However, our main finding is corroborated by the results from other studies reporting an inverse relationship between albumin and several clinical in-hospital complications as well as long lengths of stay (24) and mortality in older people (25).

In conclusion, our pilot observation in older hospitalized people suggests that measuring IL6 as well as albumin levels may help to improve risk calibration for short term all-cause mortality. It may also facilitate tailored therapeutic interventions aimed at decreasing the high mortality rate in these individuals. However, because this study enrolled a limited number of subjects, despite including several clinical and biochemical data available at baseline, our hypothesis-generating results must be validated in a larger cohort with similar characteristics.
